# Copeptin as a Diagnostic and Prognostic Biomarker in Cardiovascular Diseases

**DOI:** 10.3389/fcvm.2022.901990

**Published:** 2022-07-04

**Authors:** Danni Mu, Jin Cheng, Ling Qiu, Xinqi Cheng

**Affiliations:** ^1^Department of Laboratory Medicine, Peking Union Medical College Hospital, Peking Union Medical College and Chinese Academy of Medical Sciences, Beijing, China; ^2^State Key Laboratory of Complex Severe and Rare Diseases, Peking Union Medical College Hospital, Peking Union Medical College and Chinese Academy of Medical Sciences, Beijing, China

**Keywords:** copeptin, cardiovascular diseases, acute myocardial infarction, heart failure, biomarker

## Abstract

Copeptin is the carboxyl-terminus of the arginine vasopressin (AVP) precursor peptide. The main physiological functions of AVP are fluid and osmotic balance, cardiovascular homeostasis, and regulation of endocrine stress response. Copeptin, which is released in an equimolar mode with AVP from the neurohypophysis, has emerged as a stable and simple-to-measure surrogate marker of AVP and has displayed enormous potential in clinical practice. Cardiovascular disease (CVD) is currently recognized as a primary threat to the health of the population worldwide, and thus, rapid and effective approaches to identify individuals that are at high risk of, or have already developed CVD are required. Copeptin is a diagnostic and prognostic biomarker in CVD, including the rapid rule-out of acute myocardial infarction (AMI), mortality prediction in heart failure (HF), and stroke. This review summarizes and discusses the value of copeptin in the diagnosis, discrimination, and prognosis of CVD (AMI, HF, and stroke), as well as the caveats and prospects for the application of this potential biomarker.

## Introduction

Cardiovascular disease (CVD) is currently acknowledged as a primary threat to the health of the global population. According to the Global Burden of Diseases, Injuries, and Risk Factors Study (GBD) 2016, CVDs are attributed to the largest number of deaths among non-communicable diseases, with an estimated 17.8 million (95% uncertainty interval 17.5–18.0 million) deaths, accounting for approximately one-third of all deaths globally (1). Driven by the growth and increasing age of the population, the total global deaths from CVD increased by 21.1% between 2007 and 2017 (1, 2). Multiple complicated factors have been found to contribute to the development and progression of CVDs, including individual-level risk factors [smoking (3), elevated blood pressure (4, 5), and cholesterol (6)], as well as societal-level health determinants (2). Deaths in patients with CVD mainly result from ischemic heart disease, stroke, hypertensive heart disease, cardiomyopathy, rheumatic heart disease, and atrial fibrillation (2). Mortality and morbidity caused by CVDs can be devastating for families and cause an enormous economic burden to society. Therefore, early identification and accurate diagnosis are crucial for adequate intervention and personalized treatment of relevant patients.

**Figure 1:**
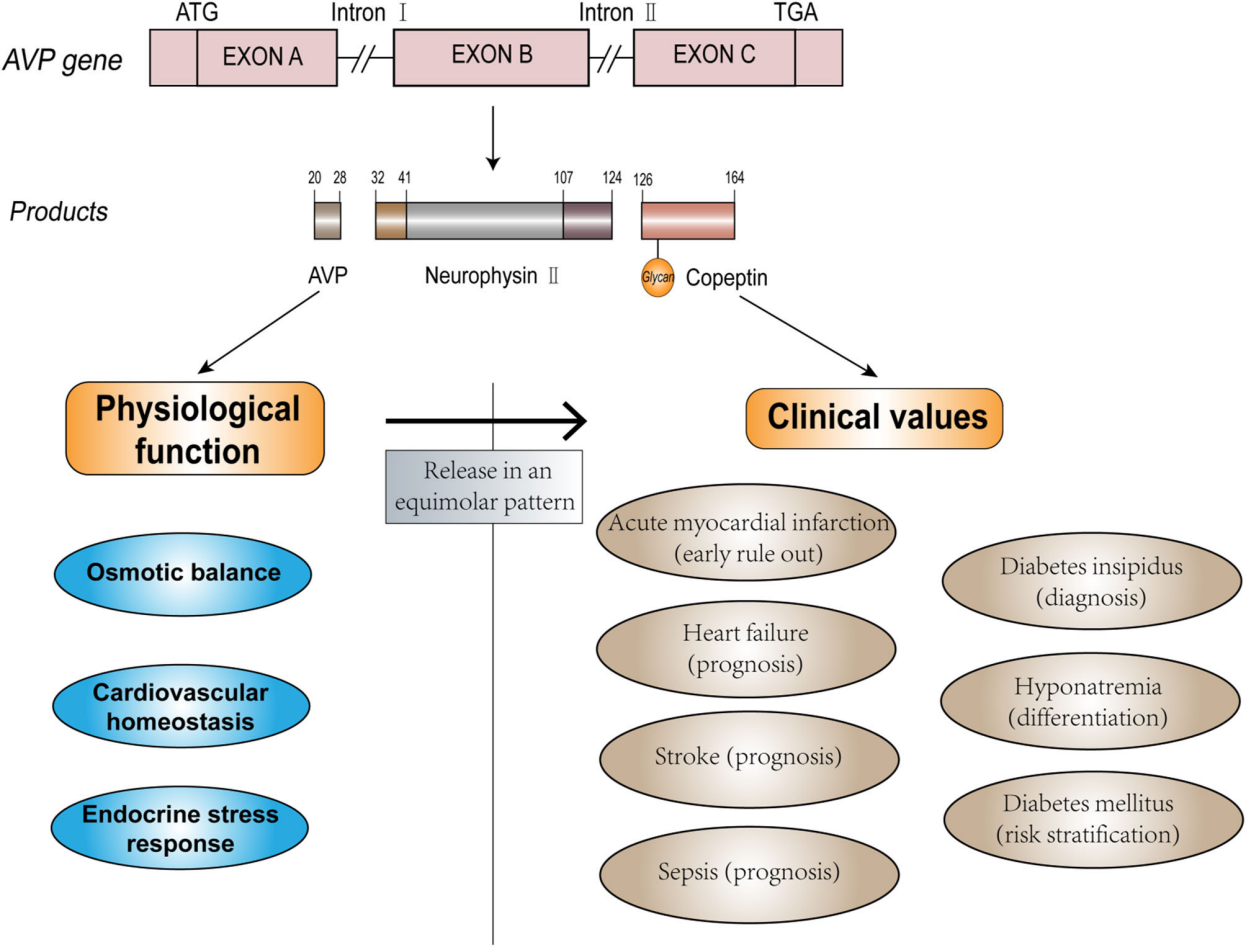
The structure, function, and clinical values of AVP and copeptin.

Arginine vasopressin (AVP) is a crucial hormone that regulates fluid homeostasis, vasoconstriction, and endocrine stress response. As the C-terminal fragment of the pro-AVP precursor, copeptin is released in an equimolar amount with AVP in response to osmotic, hemodynamic, and stress stimuli (7, 8). Copeptin has additional advantages compared to AVP (9–11) and has proven to be a potential biomarker and has been widely evaluated for its diagnostic value in clinical practice. The physiological function of endogenous AVP and potential clinical use of copeptin are depicted in Figure 1. Increased copeptin concentrations have also been described in acute disorders, including sepsis, stroke, and acute myocardial infarction (AMI) (12–14). Among these diseases, diagnosis, and management of CVDs could benefit the most from the introduction of copeptin measurement (15). Copeptin plays a variety of roles in the differential diagnosis, risk stratification, and prognostic prediction of patients with CVD, and it exerts additional value in the rapid rule-out of AMI and outcome prediction of heart failure (HF) when used in combination with other conventional cardiac biomarkers. Although copeptin has received increasing attention from medical practitioners, further investigation and evidence are required to provide better identification and differentiation of certain diseases to be accepted as a routine clinical measurement in the future. Here, we discuss the clinical efficacy of copeptin in CVDs, as well as the limitations and prospects of the clinical utility of copeptin as a routine biomarker.

## Biology of Copeptin and Vasopressin

Copeptin, a leucine-rich glycopeptide (molecular mass ~4 kDa), was first discovered by Holwerda from the posterior pituitary of pigs (7), and its 39 amino acid sequence was determined in 1981 (human: ASDRSNATQLDGPAGALLLRLVQLAGAPEPFEPAQPDAY) (16). Copeptin is derived from the precursor peptide pre-pro-vasopressin (164 amino acids), which is formed from the AVP peptide, neurophysin II, and copeptin (17, 18). AVP, also known as antidiuretic hormone (ADH), is a 9 amino acids peptide [CYFQNCPRG (disulfide bond cys1-cys6)]. The gene that encodes this precursor is located on the short arm of chromosome 20 (20p13), with three exons and two introns (19, 20).

Pre-pro-vasopressin is synthesized in the magnocellular neurons of the supraoptic nucleus (SON) and paraventricular nucleus (PVN) of the hypothalamus. The axons of these neurons constitute the hypothalamic-pituitary tract, which terminates in the posterior pituitary. During transport along the tract, the precursor is proteolytically cleaved into AVP, neurophysin II, and copeptin, and the products are stored in the posterior pituitary (21–23). The complete process of synthesis, transport, and storage takes ~1–2 h (24). Elevated osmotic pressure or decreased volume of circulating fluids can stimulate osmoreceptors in the hypothalamus, resulting in the stoichiometric release of AVP and copeptin into nearby capillaries (25). AVP and copeptin secretions are also regulated by other neuroendocrine mechanisms. The parvocellular neurons of the PVN can produce the precursor, which is transported along the portal vessel to the anterior pituitary. In this pathway, AVP can interact with corticotrophin-releasing hormone (CRH) to stimulate the release of adrenocorticotropic hormone (ACTH) from endocrine cells in the anterior pituitary, reflecting somatic stress levels (26).

After release into the bloodstream, AVP activates intracellular effectors by binding to three receptor subtypes [V1a, V1b (V3), and V2] (27, 28). V1a receptor (V1aR) is located mainly in smooth muscle vascular cells (also in the liver, brain, and platelets) and controls cell contraction. V1bR is located in the anterior pituitary (and other areas in the brain, such as the hypothalamus) and regulates the release of ACTH, which stimulates the release of cortisol in the adrenal cortex [known as the hypothalamic-pituitary adrenal (HPA) axis] (29–31). V2R exists mainly in the basolateral membrane of collecting tubules in the kidney and mediates the aquaporin-2 water channel insertion into the apical membrane of collecting duct cells, causing water reabsorption, and maintaining osmotic homeostasis (32, 33). The neurophysin II gene with a missense mutation was found to be associated with human autosomal dominant neurohypophyseal diabetes insipidus (34). However, the physiological function of copeptin is unclear, and the specific receptor for copeptin has not yet been identified. A previous study proposed that copeptin could stimulate prolactin release from cultured pituitary cells, which was not supported by another study reporting that copeptin could not serve as a prolactin-releasing factor (35, 36). Another hypothesis indicated that copeptin assisted the refolding of the precursor by facilitating the interaction of misfolded monomers with the calnexin/calreticulin system (37). However, these hypotheses have not been fully validated. Copeptin is regarded as a surrogate marker of AVP.

The release of copeptin is regulated by osmotic pressure changes (38, 39). Fenske et al. (11) showed that median osmotic pressure (289–311 mOsm/kg H_2_O) caused similar release kinetics of plasma copeptin (from 4 to 29.3 pmol/L) and AVP (from 1 to 10.3 pg/ml). They also reported that copeptin and AVP concentrations were highly correlated, with a Spearman rank correlation coefficient of 0.94. Non-specific somatic stress is another factor that regulates copeptin. Copeptin levels are significantly higher in patients with acute diseases, such as sepsis (40), AMI, stroke (41), and preeclampsia (42), and can serve as a marker of endogenous stress levels (43). However, the clearance of copeptin *in vivo* has not yet been clearly investigated. Roussel et al. (44) found that copeptin levels were inversely correlated with decreasing glomerular filtration rate in patients with chronic kidney disease, indicating an association between decreased copeptin clearance and impaired renal function.

The normal range of plasma copeptin level is relatively low, which was determined to be 4.2 pmol/L (range 1–13.8 pmol/L) in a healthy cohort of 359 individuals (9). The distribution of copeptin concentration in the healthy population is skewed, and the 95, 97.5, and 99 percentiles are 9.8, 13, and 18.9 pmol/L, respectively (45). Median copeptin levels were significantly higher in men than in women but were not correlated with age (9, 46). The circadian rhythm and nutrient intake were found to slightly influence copeptin levels (47, 48), suggesting that copeptin is a robust and reliable marker in healthy individuals. Copeptin appeared to be increased by exercise but returned to normal levels after 1 h (9). In healthy participants who received an infusion of 3% hypertonic saline, copeptin was significantly higher in those who experienced nausea and/or vomiting (median 39.0 vs. 20.0 pmol/L) (49). Increased blood pressure was reported to correlate with plasma copeptin in a large population-based study of young and healthy adults (50). Confounding factors must be identified before interpreting the results based on the copeptin levels.

AVP has several detection limitations, including a short half-life, instability in circulation, and association with platelets, and the radioimmunoassay is not readily available in routine check-ups (10, 12, 51). Copeptin, in contrast, has a longer half-life (26 vs. 12 min of AVP) and is more stable; thus, it is simpler to measure using two commercially available approaches: the Brahms original sandwich immunoluminometric assay and the immunofluorescent assay on the KRYPTOR platform (9, 11). The turnaround time is 0.5–2.5 h, with a detection limit of 0.4 and 0.69 pmol/L, respectively. There are also some ELISA assays available but only used by research, and they lack approval and clinical validation. Plasma and serum samples are suggested to be used to measure copeptin concentration, while other sample types like cerebrospinal fluid need more validation before application (52). In all, copeptin is regarded as a potential surrogate marker for AVP.

## Copeptin in AMI

As a common cardiac emergency, AMI caused by myocardial ischemia and necrosis has a high risk of morbidity and mortality if not treated timeously (53). Therefore, patients with the suspected acute coronary syndrome (ACS) should be referred immediately to the emergency department (ED) for evaluation (54). However, only ~10% of patients with chest pain in the ED were diagnosed with AMI (55), and a delay in the rapid rule-out of AMI can preclude the detection of the underlying disease, require prolonged monitoring, and serial blood sampling of patients, and waste medical resources. Besides, the “gray zone” for troponin elevation makes the sensitivity and negative predictive value (NPV) unsatisfactory. Therefore, rapid rule-out of AMI to avoid deleterious consequences for patients and elevated medical costs remains a major challenge. In addition, an effective procedure for risk stratification, as well as prompt identification and assessment of patients with AMI who are at risk of adverse outcomes, is essential for optimized care and resource allocation to preclude serious disability or sequelae. Physical examinations, such as 12-lead electrocardiography (ECG), are necessary for patients with symptoms suggestive of AMI, but the approaches can be confusing and unclear when the results are negative. Therefore, biomarkers that can rapidly and definitively reflect cardiac abnormalities are required.

Cardiac troponins (cTn) are a group of cardiac structural proteins that regulate the calcium-mediated interaction of actin and myosin (56). When a sufficient number of myocytes die due to necrosis or apoptosis, cTn can be detected in the blood (57). Two specific cardiac isoforms of troponin are cTnI and cTnT which are almost exclusively in the heart (58), but cTnT is found to express in skeletal muscles to a minor extent, which means some elevations of cTnT might be due to skeletal muscle abnormality (59). In patients with AMI, cTnI and cTnT start to appear in the circulation early after the AMI onset. The biomarkers reach a peak after 14–16 h and return to a normal level for 4–10 days (60, 61). According to the Fourth Universal Definition of Myocardial Infarction (62), AMI is defined as when there is an acute myocardial injury with clinical evidence of acute myocardial ischemia and with detection of a rise and/or fall of cTn values with at least one value above the 99th percentile URL. In 2007–2010, high-sensitivity assays started to be used to improve cTn assessment, and assays for high-sensitivity cTn (hs-cTn) with a coefficient of variance of <10% at the 99th percentile of the reference population have been developed and widely used (63). Compared with conventional cTn assays, hs-cTn assays demonstrate several advantages, including a higher NPV for AMI and reduction of the “troponin-blind” interval (to detect cTn increase earlier) (60, 64). Therefore, hs-cTn became the preferably recommended laboratory test to diagnose AMI in clinical practice (65). However, since cTn is an organ-specific instead of disease-specific biomarker, multiple factors must be considered (including age, sex, renal dysfunction, and time from chest pain onset) when interpreting the results. Besides, cTn cannot be used alone but in conjunction with careful assessment of chest pain characteristics and ECG.

Unlike cTn and other cardiac biomarkers, copeptin, which non-specifically reflects the endogenous stress level at the onset of AMI, has been advanced and widely validated for its clinical value in AMI.

### The Release Pattern of Copeptin and cTn in Patients with AMI

As a marker of endogenous stress, copeptin levels rise sharply after myocardial injury following a swift decline (45, 66). A meta-analysis, including 14 studies (9,244 patients), showed that patients with AMI had higher copeptin levels than those without AMI (22.8 vs. 8.3 pmol/L, respectively) at presentation (14). Patients with non-ST-elevation myocardial infarction (NSTEMI) had a significantly lower level of copeptin than those with ST-elevation myocardial infarction (STEMI) but a higher level than those with unstable angina, indicating that copeptin levels were strongly associated with the extent of myocardial necrosis (67).

The release pattern of copeptin in 145 patients undergoing percutaneous coronary intervention (PCI) for a first STEMI was reported by Gu et al. (61), who reported that copeptin levels increased immediately after symptom onset to a peak of 249 pmol/L and normalized within 10 h. Similarly, Slagman et al. (68) reported that copeptin levels were already increased (median 94.0 pmol/L) at the time of first medical contact in the ambulance and decreased significantly over time in patients with NSTEMI. The diagnostic performance of copeptin was very high in early presenters [area under the curve (AUC) 0.96, 95% confidence interval (CI) 0.90–1.0; NPV 100% in the ambulance], but generally decreased over time (AUC 0.75, 95% CI 0.59–0.92 at 2 h), indicating that the particular time of blood collection can affect the amount of information copeptin provided. In contrast, conventional cardiac biomarkers, including creatine kinase isoenzyme (CK-MB), cTn, and hs-cTn increased to their maximum levels at >10 h (61, 67). A comparison between the release patterns of copeptin and other markers after AMI symptom onset is shown in Table 1. Interestingly, in 34 participants who underwent experimental balloon-induced ischemia, cTnI and cTnT levels increased after 30 s of ischemia, whereas copeptin levels did not change significantly (69). The reason for this inconsistency might be that the short duration of the induced reversible ischemia failed to affect the hemodynamic vascular system sufficiently to induce vasopressin release from the hypothalamus.

**Table 1:** Comparison between the release patterns of copeptin and other markers after symptom onset.

**Biomarker**	**99th percentile in reference population**	**Elevating start time (after symptom onset)**	**Peak time (after symptom onset)**	**Peak concentration**	**Recovery time**	**References**
Copeptin	18.9 pmol/L	0 h (on admission)	0 h (on admission)	249 pmol/L	Within 10 h	(45, 61, 66)
cTnT	10 ng/L	1–2 h	14–16 h	5,750 ng/L	>16 h	
hs-cTnT	14 ng/L	1–2 h	14–16 h	4,160 ng/L	>16 h	
CK-MB	NR	1–2 h	14–16 h	275 U/L	>16 h	
cTnI	40 ng/L	0–2 h	8–10 h	1,700 ng/L	>12 h	
Myoglobin	NR	0–3 h	0–3 h	144 ng/mL	>12 h	
NT-proBNP	NR	0–3 h	6–12 h	606.5 pg/mL	>12 h	

### Copeptin to Rapidly Rule-Out AMI

For patients with AMI, the somatic stress burden is high, and the vasopressin system is activated (70). Copeptin as a surrogate marker of AVP and can indicate endogenous stress. However, as a single non-specific biomarker, copeptin showed modest diagnostic accuracy in the early rule-out of AMI [pooled sensitivity 0.67 (95% CI 0.60–0.73), pooled specificity 0.63 (95% CI 0.57–0.69)] according to a meta-analysis, including 15 studies (8,740 patients) (71), and copeptin alone should not be used as a single diagnostic marker in patients with suspected AMI, especially in late presenters (72). However, when used in combination with other classic biomarkers, copeptin evaluation has emerged as a complementary method.

#### The Dual-Marker Strategy Involving Copeptin and Cardiac Troponin

Given the different temporal release patterns of copeptin, when it is used in combination with the time-dependent cardiac marker cTn (dual-marker strategy), copeptin has the potential to allow for rapid and accurate rule-out of AMI. This hypothesis was first proposed by Reichlin et al. (67) in 2009 and was confirmed in the following trials (45, 73–77) and meta-analysis (14, 71), that the combination of copeptin and cTn significantly improved the specificity and NPV compared with cTn alone [increased sensitivity from 0.686 (95% CI 0.661–0.710) to 0.905 (95% CI 0.888–0.921), NPV from 0.93 (95% CI 0.924–0.936) to 0.97 (95% CI 0.964–0.975)] (14); thus, a higher proportion of patient discharges was achieved, preventing serial blood drawing and testing, prolonged hospital stay and surplus medical costs (64, 78). In Table 2, we summarize the published research on the diagnostic performance to rapidly rule out AMI when using cTn in combination with copeptin, suggesting that copeptin has the potential to improve the sensitivity and NPV of cTn, especially in patients presented early after chest pain onset, with a pre-specified cutoff value. The routine use of copeptin as an additional biomarker with conventional cTn assays for the early rule-out of MI is recommended by the 2020 ESC Guidelines (65).

**Table 2:** Articles about the diagnostic performance of cTn (with or without hs-cTn) combined with copeptin measurement in the rule-out of AMI.

**References**	**Sample size**	**Assay**	**Prespecified or preferable cut-off value**	**Corresponding diagnostic performance**	**Incremental value of copeptin used with cTn**
Reichlin et al. (67)	Total 487 (81 with AMI)	cTnT: Elecsys 2010, Roche Diagnostics; copeptin: LUMItest CT-proAVP, Brahms.	Copeptin level 14 pmol/L+ cTnT 0.01 μg/L	Sensitivity 98.8%, specificity 77.1%, NPV 99.7%, PPV 46.2%.	AUC from 0.86 (0.80–0.92) for cTnT alone to 0.97 (0.95–0.98).
Keller et al. (45)	Total 1,386 (299 with AMI)	cTnT: Elecsys 2010, Roche Diagnostics; copeptin: CTproAVP LIA B.R.A.H.M.S AG.	Copeptin level 9.8 pmol/L or cTnT 0.03 ng/ml;	Sensitivity 90.9% (87.1–93.9%), specificity 68.3% (65.1–71.3%), NPV 95.8% (93.9–97.2%), PPV 48.8% (44.6–53.1%).	AUC from 0.84 (0.82–0.87) for cTnT alone to 0.93 (0.92–−0.95).
Charpentier et al. (73)	Total 641 (95 with NSTEMI)	cTnI: ADVIA Centaur analyzer, Siemens Medical Solutions Diagnostics; copeptin: KRYPTOR analyzer, Brahms AG.	Copeptin level 12 pmol/L+ cTnI 0.1 μg/L	(for NSTEMI) Sensitivity 90.4% (88.2–92.7%), specificity 66.3% (62.4–70.0%), NPV 97.6% (96.4–98.7%), PPV 31.6% (28.0–35.2%).	AUC from 0.77 (0.72–0.82) for cTnT alone to 0.89 (0.85–0.92).
Potocki et al. (74)	Total 1,170 (433 with pre-existing coronary artery disease; 78 with AMI)	Fourth generation cTnT and hs-cTnT: Elecsys 2010 system, Roche Diagnostics; Copeptin: LUMItest CT-proAVP, Brahms.	Copeptin level 9 pmol/L, Roche troponin T fourth generation (cTnT) 0.01 μg/L, Roche hs-cTnT 0.014 μg/L.	(in Patients with pre-existing CAD) Copeptin + cTnT: sensitivity 98.7% (93.0–99.8), specificity 53.5% (48.2–58.8), NPV 99.5% (97.1–99.9), PPV 31.8% (26.0–38.1). Copeptin + hs-cTnT: sensitivity 98.7% (93.0–99.8), specificity 41.4% (36.2–46.7), NPV 99.3% (96.3–99.9), PPV 27.0% (22.0–32.6).	AUC from 0.86 (for cTnT alone) to 0.94; from 0.92 (for hs-cTnT alone) to 0.94.
Ray et al. (79)	Total 451 (36 with AMI)	cTnI: X-pand HM analyzer, Siemens Healthcare Diagnostics Inc; Access analyzer, Beckman Coulter, Inc,; Advia Centaur analyzer, Siemens Healthcare Diagnostics Inc; copeptin: CT-proAVP LIA B.R.A.H.M.S AG.	X-pand HM cTnI 0.07 μg/L; Access analyzer cTnI 0.04 μg/L; Advia Centaur analyzer cTnI 0.1 μg/L; copeptin 10.7 pmol/L.	Sensitivity 83% (64–96), specificity 61% (57–66), NPV 98% (95–99), PPV 14% (9–20).	AUC from 0.734 (0.670–0.791) for cTnI-ADV alone to 0.873 (0.821–0.914); AUC from 0.540 (0.458–0.620) for cTnI on Advia Centaur alone to 0.749 (0.673–0.815).
Chenevier-Gobeaux et al. (80)	Total 317 (45 with AMI)	cTnI: X-pand® HM analyser, Siemens Healthcare Diagnostics Inc; Access® analyser, Beckman Coulter, Inc.; copeptin: KRYPTOR analyzer	X-pand cTnI 0.14 μg/L; Access cTnI 0.06 μg/L; copeptin 10.7 pmol/L	Sensitivity 98% (87–100), specificity 54% (47–62), NPV 99% (97–100), PPV 26% (20–33).	Sensitivity and NPV from 71% (55–83) and 95% (92–97) for cTnI alone to 98% (87-100) and 99% (97–100).
Maisel et al. (66)	Total 1,967 (156 with AMI)	cTnI: ADVIA Centaur XP system, Siemens Healthcare Diagnostics; copeptin: KRYPTOR analyzer, Brahms AG.	cTnI 40 ng/L; copeptin 14 pmol/L	Sensitivity 92.2% (85.9–95.9), specificity 62.6% (60.4–64.8), NPV 99.2% (98.5–99.6), PPV 13.6% (11.4–16.2).	AUC from 0.86 for cTnI alone to 0.97.
Charpentier et al. (81)	Total 587 (87 with NSTEMI)	cTnI: ADVIA Centaur immunoassay system; copeptin: KRYPTOR analyzer, Brahms AG.	cTnI 0.05 μg/L, Copeptin 12 pmol/L	(for NSTEMI) Sensitivity 96.6% (90.3–99.3), specificity 65.0% (60.6–69.2), NPV 99.1% (97.4–99.8), PPV 32.4% (26.8–38.5).	AUC from 0.94 (0.91–0.97) for cTnI alone to 0.95 (0.93–0.97).
Folli et al. (82)	Total 472 (28 with STEMI; 28 with NSTEMI)	cTnT: third-generation assay; copeptin: BRAHMS AG, Henningsdorf, Germany	Copeptin 14 pmol/L; cTnT not mentioned.	(For both ACS and non-ACS), NPV 85%; (for ACS) NPV 86.6%.	(for NSTEMI) AUC from 0.76 for cTnIT alone to 0.86. (for STEMI) AUC from 0.86 for cTnI alone to 0.89.
Afzali et al. (83)	Total 230 (24 with STEMI; 83 with NSTEMI)	cTnI: ADVIA-Centaur XP system, Siemens Healthcare Diagnosis; copeptin: CT-proAVP LIA B.R.A.H.M.S AG.	cTnI 0.04 ng/mL; copeptin 14 pmol/L.	Sensitivity 85.7%, specificity 66.4%, PPV 25%, NPV 97.3%.	Not reported.
Collinson et al. (84)	Total 850 (68 with AMI)	cTnI: Siemens ultra; POCT measurement; cardiac troponin I (cTnI) Stratus CS; Beckmann; cardiac troponin T high sensitivity; copeptin: not reported.	cTnI 99th percentile; Copeptin 7.4 mg/L	Not reported.	AUC of cTnI Stratus CS 0.94 (0.90–0.98), cTnI Beckmann 0.92 (0.88–0.96), cTnI Siemens ultra 0.90 (0.85–0.95), cardiac troponin T high sensitivity 0.92 (0.88–0.96), copeptin 0.62 (0.57–0.68). The combination of troponin (at the 99th percentile) increased diagnostic sensitivity.
Vafaie et al. (85)	Total 131 (28 with NSTEMI)	cTnT: Radiometer AQT90 Flex (Radiometer POCT); Roche Cobas h232 CARDIAC T Quantitative (Cobas POCT); copeptin: KRYPTOR analyzer, Brahms AG.	Radiometer cTnT 0.017 μg/L, Cobas cTnT 14 μg/L; copeptin 10 pmol/L.	Radiometer cTnT + copeptin: Sensitivity 85.7% (0.728–0.987), specificity 66.0% (0.569–0.752), NPV 94.4% (0.892–0.997), PPV 40.7% (0.281–0.532). Cobas cTnT + copeptin: Sensitivity 89.3% (0.778–1.007), specificity 68.0% (0.589–0.770), NPV 95.9% (0.913–1.004), PPV 43.1% (0.304–0.558).	AUC from 0.822 for Radiometer cTnT alone to 0.826; AUC from 0.725 for Cobas cTnT alone to 0.814.
Ricci et al. (86)	Total 196 (29 with NSTEMI)	cTnI: Dimension VISTA cTnI assay, Siemens Healthcare Diagnostics; copeptin: KRYPTOR analyzer, Brahms AG.	cTnI 0.045 μg/L; copeptin 10 pmol/L.	(on admission) Sensitivity 100%, specificity 74.2%, NPV 100%, PPV 40.3%.	(on admission) AUC from 0.891 (0.838–0.931) to 0.871 (0.816–0.915).
Chenevier-Gobeaux et al. (87)	Total 885 (114 with AMI)	cTnI: X-Pand HM analyser, Siemens Healthcare Diagnostics Inc.; Access analyser, Beckman Coulter Inc.; Advia Centaur analyzer, Siemens Healthcare Diagnostics Inc.; copeptin: KRYPTOR analyzer, Brahms AG.	HM cTnI 0.07 μg/l; Beckman cTnI 0.04 μg/l; Advia cTnI 0.1 μg/l; copeptin 10.7/14.1 pmol/L.	(patients ≥70 years, cTnI and/or copeptin >10.7 pmol/L): Sensitivity 93% (92–98), specificity 48% (40–56), NPV 95% (87–98), PPV 38% (30–46). (patients <70 years, cTnI and/or copeptin > 14.1 pmol/L): Sensitivity 92% (81–97), specificity 75% (71–78), NPV 99% (97–100), PPV 26% (20–32)	(patients ≥70 years), sensitivity and NPV from 62 and 88% for cTnI alone to 93 and 95%.
Giannitsis et al. (88)	Total 2,294	hs-cTnT: Roche Elecsys hs-cTnT; Abbott Architect hs-cTnI; Siemens (Vista, Loci); cTnI Beckman Access TnI; Radiometer (third generation cTnT). Copeptin: KRYPTOR analyzer, Brahms AG.	cTn: 99th percentile; copeptin 10pmol/L.	All-cause mortality was 0.1% (0–0.6%) in the primary DMS discharge group versus 1.1% (0.6–1.8%) in the conventional workup group.	Copeptin on top of cTn supports safe discharge.
Jeong et al. (76)	Total 271 (43 with STEMI; 25 with NSTEMI)	cTnI: Advia, Centaur XP, Siemens Healthcare Diagnostics Inc.; copeptin: KRYPTOR analyzer, Brahms AG.	cTnI 0.78 μg/L; copeptin 10 pmol/L.	(chest pain onset ≤ 1 h) Sensitivity 90.24% (76.87–97.28), specificity 60.38% (50.41–69.75), NPV 94.12% (86.16–97.63), PPV 46.84% (40.55–53.22).	(for AMI, chest pain onset ≤ 1 h) AUC from 0.719 for cTnI to 0.753.
Giannitsis et al. (89)	Total 10,329	cTn: NR; hs-cTnT: Cobas e411; Elecsys 2010, Roche Diagnostics; hs-cTnI: Abbott Arcitect STAT assay; copeptin: KRYPTOR analyzer, Brahms AG.	cTn: NR; hs-cTnT 5 ng/L; hs-cTnI 2 ng/L; copeptin 10/14 pmol/L.	Sensitivity 94.9% (91.7–97.8%), NPV 99.4% (99.02–99.64%).	(for NSTEMI) NPV from 97.6% (97.2–98.0%) for cTn alone to 98.6% (98.2–99.0%); NPV from 98.8% (98.4–99.1%) for hs-cTn to 99.4% (99.0–99.6%).
Ahmed et al. (90)	Total 90 (39 with NSTEMI)	cTnI: Architect I 1000 (Abbott Diagnostics, USA); copeptin: double-antibody sandwich ELISA kit (Sunredbio, Shanghai).	cTnI 0.07 ng/ml; Copeptin 2.34 ng/ml	(For NSTEMI) Sensitivity 100%, specificity 93%, NPV 100%, PPV 93.5%.	AUC from 0.888 (0.819–0.956) for cTnI alone to 0.975 (0.944–1.000).

#### The Incremental Value of Copeptin Combined with hs-cTn

Although the combination of copeptin with conventional cTn provided significant benefits, the incremental value of copeptin when used with hs-cTn compared with hs-cTn alone remains unclear and inconsistent for an instant the rule-out of NSTEMI (75, 91–93). Several studies evaluated whether adding copeptin (with a pre-specified cut-off value) could increase the rule-out performance of hs-cTn (with a cut-off of 99th percentile of the healthy population) on admission with only single blood collection, and reported only numerically small or no incremental value (94–98). Therefore, it is suggested that copeptin could be effective only used with less sensitive conventional cTn assays, but not with hs-cTn (64, 65).

When the hs-cTn assay is available and well validated, patients with “very low” or limit of detection (LOD) level of hs-cTn (Roche hs-cTnT < 5 ng/L; Abbott hs-cTnI < 4 ng/L) on admission (0 h) could be eligible for “rule-out” and may be discharged if the ECG and/or clinical symptoms suggest a low risk of ACS (65). In order to compared the “very low” algorithms with combination of copeptin level assessment on admission, Restan et al. (99) recently reported that adding copeptin < 9 pmol/L did not increase the sensitivity of the rule-out provided by hs-cTnT < 5 ng/L or hs-cTnI < 4 ng/L in patients presenting > 3 h after chest pain onset (sensitivity 98.9% for hs-cTnT < 5 ng/L vs. 98.9% for hs-cTnT < 7 ng/L and copeptin < 9 pmol/L, NPV 99.6 vs. 99.7%; sensitivity 97.8% for hs-cTnI < 4 ng/L vs. 97.8% for hs-cTnI < 7 ng/L and copeptin < 9 pmol/L, NPV 99.5 vs. 99.5%). Interestingly, when analyzing patients presenting early (<3 h) from chest pain onset, higher sensitivity for the dual marker strategy was observed in one cohort. This is consistent with the guidelines in which the use of a 0 h hs-cTn-only approach is not recommended (65). The reason why the incremental value of copeptin is smaller when used with hs-cTn than with cTn may be that the highly sensitive assay has, to a large extent, overcome the sensitivity deficit of conventional cTn in clinical practice, recognizing the elevation of cTn in AMI patients earlier. Therefore, the added value brought by combining copeptin into hs-cTn is much less obvious.

A secondary analysis (100) from a multicenter study, including 1,439 patients, investigated whether a second copeptin measurement (at 1 h) could improve the rule-out and rule-in algorithm for patients with initially negative hs-cTn and copeptin results. It was found that 1 h-copeptin did not significantly increase the NPV [98.6% (95% CI 97.4–99.3%) vs. 98.6% (95% CI 97.3–99.3%)], but 1 h-hs-cTnT did [NPV 99.6% (95% CI 98.7–99.9%)]. Besides, in the intermediate-risk group (negative hs-cTnT but increased copeptin on admission), a similar finding was observed. These results extended and corroborated previous findings that the concentration of copeptin had already declined, whereas that of cTn kept rising in a large proportion of AMI patients presented to the ED. In addition, it is also suggested that if the purpose is to investigate the diagnostic performance of copeptin in early rule-out AMI at admission, blood samples must be drawn as early as possible after the onset of symptoms. Blood samples were drawn several hours after admission and after treatment initiation was unqualified for this purpose (101).

However, some studies have concluded that this dual marker strategy (combination of copeptin and hs-cTn) might provide higher eligibility or efficacy (quantified by the percentage of the overall cohort assigned to the rule-out zone) to rule out NSTEMI (89, 102, 103). Giannitsis et al. evaluated pooled data of 10,329 patients and concluded that the NPV for ruling out NSTEMI was high for the dual marker strategy (copeptin < 10 pmol/L and/or hs-cTn ≤ 14 ng/L) and single marker strategy (hs-cTnT < 5 ng/L) to be 99.0 and 99.2%, respectively, but the former had a 2.4-fold higher eligibility [64.6% (63.0–66.2%) vs. 27.9% (26.2–29.7%)]. Another prospective study enrolling 1,920 patients found that the NPV (reflecting safety) was very high and comparable in the LOD algorithm (hs-cTnT < 5 ng/L) and the dual marker strategy (copeptin < 9 pmol/L and/or hs-cTn ≤ 14 ng/L) [NPV 99.6% (98.6–99.9%) vs. 98.8% (97.9–99.4%)]. For efficacy, the dual marker strategy performed better than the LOD algorithm (allowed rule-out in 47.6% of patients vs. 25.8% of patients). Using the hs-cTnI assay had similar results (102). These studies corroborated the safety and effectiveness of the dual marker strategy using hs-cTn and copeptin. However, given that more non-MI patients might be ruled in on admission and the cost of running the copeptin measurement platform 24/7, health economic analysis using the dual marker strategy vs. the single marker strategy in patients with suspected AMI in ED should be conducted, and detailed practical guidelines are desirable. Table 3 shows articles on the diagnostic performance of hs-cTn combined with copeptin measurement in the rule-out of AMI.

**Table 3:** Articles about the diagnostic performance of hs-cTn combined with copeptin measurement in the rule-out of AMI.

**References**	**Sample size**	**Assay**	**Prespecified or preferable cut-off value**	**Corresponding diagnostic performance**	**Incremental value of copeptin used with hs-cTn**
Giannitsis et al. (91)	Total 503 (49 with STEMI; 87 with NSTEMI)	hs-cTnT: Roche Diagnostics; copeptin: KRYPTOR analyzer, Brahms AG.	(prespecified) hs-cTnT 14 ng/L and copeptin 14 pmol/L	Sensitivity 97.8% (93.7–99.5%), specificity 55.9% (50.6–61.0%), NPV 98.6% (95.8–99.7%), PPV 45.1% (39.3–51.0%.	No added benefit of copeptin + hs-cTnT, compared with hs-cTnT alone to rule out non-STEMI (data not shown).
Meune et al. (94)	Total 58 (13 with AMI)	hs-cTnT: Roche Diagnostic; copeptin: KRYPTOR analyzer, Brahms AG.	hs-cTnT 14 ng/L or copeptin 14 pmol/L	(on admission) Sensitivity 86.7%, specificity 70.4%, NPV 82.6%, PPV 76.5%.	AUC 0.90 (0.81–0.99) for hs-cTnT measured on admission to 0.94 (0.88–1.00) for hs-cTnT + copeptin measured on admission.
Lotze et al. (104)	Total 142 (13 with AMI; 9 with STEMI, 4 with NSTEMI,)	hs-cTnT: Elecsys® troponin T high-sensitive; cobas® e 601, Roche Diagnostics; fourth-generation troponin T assay, Roche Diagnostics; copeptin: BRAHMS AG, Henningsdorf, Germany	hs-cTnT 14 ng/L or copeptin 14 pmol/L	Sensitivity 100%, specificity 34.9%, PPV 13.4%, NPV 100%.	Sensitivity and NPV from 92.3 and 98.6% for hs-cTnT alone to 100 and 100%.
Karakas et al. (105)	Total 366 (8 with AMI)	hs-cTnT: Elecsys 2010, Roche Diagnostics; copeptin: CT-proAVP LIA B.R.A.H.M.S AG.	hs-cTnT 13.0 ng/L, copeptin 7.38 pmol/L	No combined performance was reported.	AUC from 0.795 (for hs-cTnT alone) to 0.771.
Sebbane et al. (106)	Total 194 (52 with AMI; 25 with NSTEMI)	hs-cTnT: Cobas 8000/e602 analyzer, Roche Diagnostics; us-copeptin: Kryptor Compact Plus system, Thermofisher Scientific; copeptin: KRYPTOR analyzer, Brahms AG.	hs-cTnT 18.0 ng/L, copeptin 13.11 pmol/L	(for AMI) Sensitivity 96.2% (86.8–99.5), specificity 64.8% (56.3–72.6), PPV 50% (39.8–60.2), NPV 97.9% (92.5–99.7). (for NSTEMI) Sensitivity 96% (79.6–99.9), specificity 64.8% (56.3–72.6), PPV 32.4% (22–44.3), NPV 98.9% (94.2–100).	AUC from 0.886 (0.85–0.992) for hs-cTnT alone to 0.928 (0.89–0.967).
Thelin et al. (107)	Total 478 (70 with NSTEMI)	hs-TnT: Roche high sensitivity troponin T; copeptin: KRYPTOR analyzer, Brahms AG.	hs-cTnT 14 ng/L, copeptin 14 pmol/L	(for NSTEMI) Sensitivity 96% (87–98), specificity 49% (44–54), PPV 24% (19–30), NPV 99% (95–99).	(For NSTEMI) sensitivity and NPV from 69% (59–77) and 89% (84–92) for hs-cTnT alone to 96% (86–98) and 99% (95–99).
Bahrmann et al. (108)	Total 306 (38 with NSTEMI)	hs-cTnT: cobas e411 system, Roche Diagnostics; copeptin: KRYPTOR analyzer, Brahms AG.	hs-cTnT 0.014 μg/L, copeptin 14 pmol/L	(for NSTEMI) Sensitivity 100% (91–100), specificity 23% (18–29), PPV 16% (11–21), NPV 100% (94–100).	(for NSTEMI) AUC from 0.82 (0.75–0.89) for hs-cTnT alone to 0.83 (0.76–0.90).
Collinson et al. (96)	Total 850	hs-cTnT: Elecsys® 2010 system, Roche Diagnostics; cTnI Ultra: ADVIA Centaur® XP system; TnI: Access 2 system; copeptin: KRYPTOR analyzer, Brahms AG.	hs-cTnT 14 ng/l; cTnI Ultra 40 ng/; copeptin 17.4 pmol/l (19.1 pmol/l male, 12.9 pmol/l female)	(for Roche hs-cTnT + copeptin): sensitivity 0.841 (0.727–0.921), specificity 0.596 (0.561–0.631), NPV 0.978 (0.969–0.989).	Copeptin measurement is not recommended.
Duchenne et al. (109)	Total 102 (8 with NSTEMI)	hs-cTnT: Dimension VISTA, Siemens Healthcare Diagnostics; Copeptin: KRYPTOR analyzer, Brahms AG.	hs-cTnT 0.045 μg/L, copeptin 12 pmol/L	For NSTEMI, copeptin <12 pmol/L: sensitivity 12.5%, specificity 74.5%, PPV 4%, NPV 90.9%.	Copeptin does not add a diagnostic value at admission to ED for patients with suspected ACS without STEMI and with hs-cTnT below the 99th centile.
Bohyn et al. (110)	Total 247 (39 with NSTEMI)	hs-TnT: MODULAR ANALYTICS E170 detector, ROCHE Diagnostics; copeptin: KRYPTOR analyzer, Brahms AG.	hs-cTnT 14 ng/L, copeptin 14.1 pmol/L	Sensitivity 90% (79–96), specificity 53% (46–60), PPV 33% (26–41), NPV 95% (90–98).	Sensitivity and NPV from 72% (58–93) and 92% (88–95) for hs-cTnT alone to 90% (79–96) and 95% (90–98).
Zellweger et al. (92)	Total 379 (124 with AMI)	cTnT and hs-cTnT: Elecsys 2010 system, Roche Diagnostics; copeptin: LUMItest CT-proAVP, Brahms.	cTnT 10 ng/l, hs-cTnT 14 ng/l, copeptin 9 pmol/l	cTn + copeptin AUC: 0.86 (0.81-0.91; hs-cTn + copeptin AUC: 0.90 (0.87–0.93).	AUC from 0.79 (0.73–0.86) for cTn alone to 0.86 (0.81–0.91); AUC from 0.90 (0.86–0.93) for hs-cTn alone to 0.90 (0.87–0.93).
Hillinger et al. (100)	Total 1,439 (267 with AMI)	hs-cTnT: Elecsys 2010, Roche Diagnostics; copeptin: LUMItest CT-proAVP, Brahms.	hs-cTnT 14 ng/l; copeptin 10 pmol/L	NPV 98.6% (97.4–99.3%).	1-h copeptin did not increase the NPV significantly.
Wildi et al. (93)	Total 1,929 (358 with NSTEMI)	hs-cTnI: Dimension Vista® 1500 immunoassay system, Siemens; Access 2 analyzer Beckman Coulter; Architect system, Abbott Diagnostics; s-cTnI: Architect system, Abbott Diagnostics; ADVIA Centaur immunoassay system, Siemens; Access 2 analyzer, Beckman Coulter; copeptin: LUMItest CT-proAVP, Brahms.	Siemens hs-cTnI 0.009 μl/L; Beckman hs-cTnI 0.009 μl/L; Abbott hs-cTnI 0.026 μg/L; Abbott s-cTnI 0.028 μg/L; Siemens s-cTnI 0.04 μl/l; Beckman s-cTnI 0.04 μl/l; copeptin 9 pmol/L	(Siemens hs-cTnI +copeptin) Sensitivity 92.9% (88.1–96.1%), specificity 72.5% (69.4–75.4%), NPV 98.1% (96.7–99.0%), PPV 40.3% (35.6–45.2%).	Copeptin significantly increased AUC for two (33%) s-cTnI assays, sensitivity and NPV for all six cTn assays (100%).
Stallone et al. (111)	Total 519 (102 with AMI)	hs-cTnT: Elecsys 2010, Roche Diagnostics; copeptin: CT-proAVP LIA B.R.A.H.M.S AG; KRYPTOR analyzer, Brahms AG.	hs-cTnT 14 ng/l; copeptin 9 pmol/L	Symptom onset within 2 hours: sensitivity 91.2% (84.0–95.9), specificity 51.8% (46.9–56.7), NPV 96.0% (92.5–98.2), PPV 31.6% (26.4–37.3). Symptom onset from second hour: sensitivity 98.6% (96.5–99.6), specificity 56.8% (54.0–59.7), NPV 99.4% (98.5–99.8), PPV 35.3% (32.0–38.8).	No increase in AUC (0.87 for hs-cTnT alone to 0.86). NPV from 93% (90–95%) for hs-cTnT alone to 96% (93–98%).
Sörensen et al. (112)	Total 1,673 (280 with NSTEMI)	hsTnI: ArchitectSTAT high sensitive troponin, Abbott Diagnostics; copeptin: CT-proAVP LIA B.R.A.H.M.S AG.	hsTnI 21.7 ng/L, copeptin NR	(on admission) NPV 100% (97–100%).	(Atrial fibrillation) AUC from 0.97 for hsTnI alone to 0.98; (no atrial fibrillation) AUC from 0.96 for hsTnI alone to 0.97. No clinically relevant improvement.
Stengaard et al. (113)	Total 962 (178 with AMI)	hs-cTnT: Roche Diagnostics GmbH; copeptin: KRYPTOR analyzer, Brahms AG.	hs-cTnT 14 ng/L, copeptin 9.8 pmol/L	Sensitivity 96% (91–98), specificity 45% (42–49), NPV 98% (96-99), PPV 28% (25–32).	AUC from 0.81 for hs-cTnT alone to 0.85.
Boeddinghaus et al. (114)	Total 1,356 (39 with AMI)	hs-cTnT: Roche Elecsys 2010 high-sensitivity troponin T, Roche Diagnostics; hs-cTnI: ARCHITECT STAT high-sensitivity troponin I, Abbott Laboratories, IL, USA; copeptin: LUMItest CT-proAVP, Brahms	hs-cTnT 14 ng/L, hs-cTnI 26.2 ng/L; mild hs-cTn elevations: 26.2–75 ng/L for hs-cTnI, and 14–50 ng/L for hs-cTnT.	NR	The additional use of 1h-hs-cTnI changes, but not of copeptin, improved diagnostic accuracy of hs-cTnI at presentation.
Mueller-Hennessen et al. (103)	Total 922	hs-cTnT: Elecsys® Troponin T high sensitive, Roche Diagnostics; cTnI Ultra: ADVIA Centaur immunoassay system, Siemens Healthcare; copeptin: KRYPTOR analyzer, Brahms AG.	hs-cTnT 14 ng/L; cTnI Ultra 40 ng/L; copeptin 10 pmol/L	Sensitivity 94.8% (90.0–97.7), specificity 61.1% (57.5–64.5), NPV 98.3% (96.7–99.3), PPV 32.8% (28.5–37.4).	AUC from0.92 (0.90–0.94) for hs-cTnT alone to 0.93 (0.91–0.95).
Chenevier-Gobeaux et al. (115)	Total 449 (54 with NSTEMI)	hs-cTnT: Elecsys2010 analyser, Roche Diagnostics; copeptin: KRYPTOR analyzer, Brahms AG.	hs-cTnT 14 ng/L; copeptin 12 pmol/L	(Chest pain < 2h): Sensitivity 93% (66–100), specificity 54% (46–62), NPV 99% (93–100), PPV 18% (10–29).	(Chest pain < 2 h): AUC from 0.853 (0.789–0.904) for hs-cTnT alone to 0.897 (0.840–0.940).
Kim et al. (98)	Total 316 (28 with AMI)	hs-cTnI: ARCHITECT STAT High Sensitive Troponin-I assay, Abbott Laboratories; copeptin: KRYPTOR analyzer, Brahms AG.	hs-cTnI 26.2 ng/L; copeptin 10 pmol/L	(for NSTEMI) Sensitivity 100% (87.7–100), specificity 68.1% (61.7–74.0), NPV 100% (97.7–100), PPV 27.2% (18.9–36.8).	The NPV of the multi-marker strategy was 100% (97.7–100%), which was not inferior to that of serial hs-cTnI measurements.
Restan et al. (99)	Total 959 (124 with NSTEMI.	hs-cTnT: Roche Diagnostics hs-cTnT assay; hs-cTnI: Abbott Diagnostics hs-cTnI assay; copeptin: KRYPTOR analyzer, Brahms AG.	hs-cTnT 7 ng/L; hs-cTnI 7 ng/L; copeptin 9 pmol/L	(for NSTEMI, Chest pain onset < 3 h) Sensitivity 100% (90.0–100), specificity 42.0% (34.5–49.7), NPV 100%, PPV 25.7% (23.4–28.2).	Adding copeptin to hs-cTnT/I did not improve AUC [hs-cTnT/copeptin 0.91 (0.89–0.93) vs. hs-cTnT alone 0.91 (0.89–0.93)]; hs-cTnI/copeptin 0.85 (0.82–0.87) vs. hs-cTnI alone 0.93 (0.91–0.95).
Giannitsis et al. (116)	Total 10,329 (976 with NSTEMI)	hs-cTnT: Roche Diagnostics; copeptin: KRYPTOR analyzer, Brahms AG.	hs-cTnT 14 ng/L; Copeptin 10 pmol/L.	(For NSTEMI) NPV 99.4% (98.9–99.6), sensitivities 96.2% (93.8–97.7).	Comparably safe and efective instant rule-out with copeptin + hs-cTnT.

#### The Diagnostic Performance of Copeptin in Different Subgroups

The diagnostic performance of copeptin in different subgroups of the population was investigated further. A study on sex-based differences of copeptin combined with cTn for early rule-out of NSTEMI reported that men had a similar NPV (100%) as women [99.6% (95% CI 98.8–100%)] (145). Another study also concluded that the diagnostic performance (assessed by the AUC) of cTnT/hs-cTnT and copeptin performed, as well was in women as in men (75). Identifying NSTEMI in elderly patients is also a diagnostic challenge. A study has shown that an optimal copeptin cut-off value was 8.1 pmol/L in patients < 70 years and 10.7 pmol/L in patients > 70 years, and copeptin in combination with cTnI could also improve sensitivity and NPV for the diagnosis of NSTEMI in older patients (87). As for hs-cTn, the additional use of copeptin (>14 pmol/L) might help reliably rule-out NSTEMI (NPV 100%) in older populations presenting to the ED (108). Between different races (black population vs. Caucasian population), copeptin at a cut-off value of 14 pmol/L was reported to demonstrate an unequal diagnostic performance in ruling out AMI [NPV 98.0% (95% CI 96.2–99.1%) in the black population; 94.1% (95% CI 92.1–95.7%) in Caucasian population], which might provide more precise patient management (146).

#### Copeptin Increases the Performance of POCT Assays for cTn

Point-of-care testing (POCT) assays for cTn are important detection tools when commercial cTn or hs-cTn assays in laboratory platforms are unavailable, but the analytical sensitivity of POCT assays cannot fulfill this requirement. Vafaie et al. (85) found that the addition of copeptin increased the sensitivity of POCT assays (from 67.9 to 89.3% on Cobas and from 71.4 to 85.7% on Radiometer), indicating that copeptin could improve the diagnostic performance (sensitivity) of POCT assays, and suggested that copeptin combined with POCT for cTn might achieve a performance comparable to that of hs-cTn at admission. However, studies on the diagnostic value improvement of copeptin with POCT assays for cTn is still lacking and require further exploration and validation.

#### Caveats and Caution

Of note, there are several caveats and cautions when using copeptin to rapidly identify AMI. First, as a relatively novel biomarker, the optimal cut-off value of copeptin should be carefully investigated to reach a consensus, as assays for both copeptin and cTn have been improved to an increased sensitivity level. Many studies adopted a copeptin cut-off of 14 pmol/L (66, 67, 83, 95), mainly because it was used in the first publication (67). Keller et al. (45) compared the 95, 97.5, and 99 percentiles of the general population as the copeptin cut-off to evaluate the NPV and found the highest NPV was determined at a cut-off value of 9.8 pmol/L (95th percentile). Therefore, a lower cut-off value of 10 pmol/L should be considered to improve the NPV for the diagnosis of AMI, as recommended by the 2015 ESC guidelines (64, 77, 147).

Second, it is essential to realize that negative copeptin results do not indicate the absence of coronary artery disease, because the extent of copeptin release triggered by ischemia (such as unstable angina) was weaker than other acute stimuli, such as AMI (45, 67), and additional evidence, such as coronary angiography, is required for diagnosis (148). In addition, the copeptin/troponin ratio cannot predict the final infarct size or myocardial salvage index in patients with STEMI (149). Other available clinical check-ups and evidence, such as chest pain characteristics and ECGs, are indispensable for ruling out AMI, together with this dual marker strategy (62, 148, 150).

Third, as a non-specific biomarker, copeptin can be significantly elevated in many fluid disorders and stress-associated diseases (151–153). Elevated copeptin levels are present in approximately one in five patients with non-cardiac chest pain and are associated with aging, cardiac and non-cardiac comorbidities, and mortality (154). Therefore, in patients with suspected non-cardiac causes of acute chest pain (such as musculoskeletal pain, anxiety disorder, and gastroesophageal reflux), the increasing level of copeptin does not warrant any specific evidence of cardiac disorders and should be meticulously checked with further specific evaluations (148).

### Copeptin to Discriminate Between Type 1 and 2 Myocardial Infarction

There are different subtypes of myocardial infarction (MI), the most frequent are type 1 MI (T1MI) (caused by atherothrombotic coronary artery disease) and type 2 MI (T2MI) (associated with a mismatch between oxygen supply and demand) (62). The differentiation between T1MI and T2MI is based on the results of angiography indicating plaque disruption, and on the causes of symptoms, such as arrhythmias and severe hypertension (155). Identification and discrimination between the two subtypes may aid in proper and specific treatment, but it remains an unmet need and challenge (65). A clinical trial conducted in 2017 developed a diagnostic model (including female sex, not having radiating chest pain, and a baseline hs-cTnI ≤ 40.8 ng/L) to predict T2MI, and only achieved an AUC of 0.71 (patients with the highest score of 3 had a 72% probability of T2MI, while 5% probability with a score of 0) (156).

In 2020, a retrospective study (157) analyzed 2,071 patients in the Copeptin Helps in the Early Detection of Patients with Acute Myocardial Infarction (CHOPIN) study and investigated the discrimination ability of several biomarkers, including cTnI, copeptin, and mid-regional pro-atrial natriuretic peptide (MR-pro-ANP). They found that patients with T1MI had higher levels of cTnI at presentation and with subsequent measurements, while copeptin levels were higher in T2MI at the fourth and fifth follow-up time points but not at earlier measurements. Combining all biomarkers (including copeptin) resulted in a similar accuracy to a model using clinical variables (including sex, race, atrial fibrillation, warfarin, and location of pain) and cTnI (0.854 vs. 0.884), while adding all biomarkers to the clinical model yielded the highest AUC (0.917). Although copeptin's ability to predict T2MI was weak, its addition to the model improved discrimination by both net reclassification improvement (NRI) [0.687 (95% CI 0.434–0.940)] and integrated discrimination improvement (IDI) [0.066 (95% CI 0.031–0.101)]. Another recent study (158), however, argued discriminating between T2MI and T1MIs could be directly improved by copeptin [AUC of hs-cTnI alone 0.74 vs. 0.81 after addition of copeptin evaluation, odds ratio (OR) of copeptin in multivariable logistic regression 1.97, *p* = 0.0016]. The difference between the two studies may be attributed to the adjudication of the final diagnosis: the former is based on cTnI and the latter is based on hs-cTnI, and a higher percentage of minor T2MIs might be identified with hs-cTnI.

### Copeptin to Predicting Mortality and Adverse Outcome in AMI

Prompt assessment and identification of patients with AMI who are at risk of adverse outcomes are necessary for optimized care and resource allocation. Therefore, prognostic markers for predicting the mortality rate and adverse outcomes of MI are extensively favored. Nowadays, serial measurement of hs-cTn, as well as B-type natriuretic peptide (BNP) or N-terminal pro-B-type natriuretic peptide (NT-proBNP) concentration have been recommended for the assessment of prognosis (159, 160). Elevated hs-cTn levels were reported to predict a higher risk of death. However, the role of copeptin in the prognosis is less validated and has not been recommended.

Several studies have indicated that copeptin is a useful prognostic predictor for adverse outcomes in patients with ACS (83, 161–164), and the predictive performance for 180-day mortality was significantly better in combination with copeptin and cTnI than with cTnI alone (75). However, some controversial results have argued that there is no significant predictive ability of copeptin (alone or with cTn) for ACS outcomes (108, 165, 166). To obviate the influence of different study designs and limited sample sizes, a meta-analysis (167) was conducted to evaluate the prognostic value of copeptin in predicting mortality in ACS and found that the pooled sensitivity and specificity were 0.77 (95% CI 0.59–0.89) and 0.60 (95% CI 0.47–0.71), respectively, and the AUC was 0.73 (95% CI 0.69–0.77), indicating an acceptable performance for predicting mortality in ACS. Previous studies (166, 168, 169) have shown that a multiple-marker approach seems to provide more prognostic value for adverse outcomes in ACS. While there is a lack of evidence regarding to the incremental value of copeptin added to other multi-markers in ACS, further studies are needed to provide insight into copeptin as a prognostic marker.

For AMI patients who successfully underwent PCI, the prognostic value of copeptin was also studied, and it was reported that the increased copeptin levels were correlated with increased incidence of major adverse cardiac events (MACE) during long-term follow-up (median 30.1 months) in the participants (OR = 1.6) (170). For patients undergoing non-cardiac surgery, several studies have investigated the incremental value of copeptin in predicting MACE when added to established preoperative risk indices. Jarai et al. reported that a copeptin level >14 pmol/L was a significant independent predictor of perioperative and postoperative outcomes in patients undergoing vascular surgery (hazard ratio, HR = 2.842, *p* = 0.002) (171). Another study found that copeptin ≥ 9.6 pmol/L was associated with significantly higher rates of myocardial injury and improved risk stratification. However, current data are insufficient to provide results on the added predictive performance of copeptin for any of the outcomes, so further research is still required to improve the prognostic strategies in this group of patients (172).

## Copeptin in Heart Failure

Heart failure (HF), resulting from an abnormality in cardiac structure or function, is one of the most common causes of hospitalization and mortality worldwide (173). HF is defined as “a complex clinical syndrome that results from any structural or functional impairment of ventricular filling or ejection of blood” according to the 2013 ACCF/AHA guideline (174). A wide range of etiologies can result in HF conditions, primarily including four underlying conditions: ischemic heart disease (IHD), chronic obstructive pulmonary disease, hypertensive heart disease, and rheumatic heart disease (175). IHD was the leading cause of death globally, accounting for 8.9 million all-age deaths in 2017 (1). IHD refers to an imbalance between myocardial energy state and coronary blood flow, which can occur under the following conditions: atherosclerosis, coronary microvascular dysfunction, inflammation, and vasospasm (176). Hypertensive heart disease is ascribed to chronic blood pressure overload, which exposes cardiac myocytes to higher hemodynamic stress and neurohormones, leading to left ventricular hypertrophy. Progressive hypertrophy and fibrotic changes ultimately result in diastolic heart failure (177).

HF can be classified into two categories based on systolic function: heart failure with reduced ejection fraction (HFrEF) and heart failure with preserved ejection fraction (HFpEF). HFrEF is defined as a clinical diagnosis of HF and left ventricular ejection fraction (LVEF) < 40%, whereas HFpEF refers to those with LVEF ≥ 50% (178). Patients with LVEF in the range of 40–49% represent a “gray area”, which is defined as HF with mid-range EF (HFmrEF). Compared with HFrEF, HFpEF patients are often older women with a history of hypertension and atrial fibrillation (179). Of note, the diagnosis of HFpEF is more challenging than that of HFrEF, because HFpEF generally do not have a dilated left ventricular, making additional examination and serum biomarker tests necessary.

The clinical syndromes and signs of HF are non-specific; therefore, careful history taking and physical examination are pivotal in the assessment of HF (180, 181). Currently, BNP, NT-proBNP, and MR-pro-ANP have been recommended and extensively accepted as biomarkers for the evaluation and differentiation of patients presenting in the ED or CCU/ICU with symptoms suggestive of HF (178, 182, 183). Patients with BNP < 100 pg/ml, NT-proBNP <300 pg/ml, and MR-proANP < 120 pg/ml can be excluded from HF diagnosis (178). The diagnosis of HFpEF is more difficult because other potential non-cardiac causes of symptoms suggestive of HF are supposed to be excluded. A recent meta-analysis evaluated the diagnostic accuracy of currently available biomarkers for chronic HFpEF and showed that the pooled sensitivities and specificities of BNP and NT-proBNP were 0.787 (95% CI 0.719–0.842) and 0.796 (95% CI 0.672–0.882) for BNP, and 0.696 (95% CI 0.599–0.779) and 0.882 (95% CI 0.778–0.941) for NT-proBNP, respectively (184). Although BNP and NT-proBNP remain the most reliable biomarkers for HFpEF diagnosis, it is worth noting that elevated levels of natriuretic peptides can be non-specific and caused by a series of cardiac and non-cardiac factors, including pulmonary embolism, myocarditis, and renal dysfunction (185, 186). Therefore, additional biomarkers and improved treatment strategies are required.

### Vasopressin System in HF

Owing to a relatively well-established knowledge, the current treatment of HF is mainly targeted at the neurohormonal systems with a predominance of a blockade of adrenergic receptors and the renin-angiotensin-aldosterone system, such as angiotensin-converting enzyme inhibitors, beta-blockers, and angiotensin-II receptor antagonists (178). Overactivation of the vasopressin system in patients with HF has been largely neglected (187).

Patients with HF suffer greatly from water retention and volume overload, which is assumed to be attributed to the inappropriate activation of the vasopressin system. In addition, a non-osmotic pathway is also believed to regulate AVP release through intra-cardiac pressures, intra-arterial pressures, angiotensin II, pain, and adrenergic central nervous stimuli (25, 188). Significantly increased AVP levels have been reported in patients with HF (acute HF with hyponatremia and chronic HF with or without hyponatremia) and LV dysfunction (25, 189–194). Regarding the pharmacological value, vasopressin receptor antagonists can block the V2R in renal tubules to promote aquaresis or can block the V1aR in smooth blood vessels to inhibit vasoconstriction and cardiac remodeling (195). Tolvaptan, a highly selective vasopressin V2 receptor antagonist, may be used to treat patients with volume overload and resistant hyponatremia; however, it was found to have no effect on long-term mortality or HF-related morbidity in patients hospitalized with HF (196).

### Clinical Value of Copeptin in HF

The performance of copeptin as a surrogate marker of AVP in risk stratification of patients with HF has been proposed and widely evaluated. Xu et al. found (197) that copeptin and NT-proBNP levels increased as NYHA grade increased in patients with HFrEF, but not in patients with HFpEF. In addition, increased copeptin levels in patients with advanced HF have also been reported to be associated with a reduced cardiac index (138). These results, together with other evaluations (119, 198), suggest the clinical value of risk stratification and disease severity of copeptin in patients with HF. Furthermore, in a prospective cohort study (143), individuals in the top quartile of copeptin had a significantly increased risk of developing HF (HR = 1.63, 95% CI 1.20–2.21) compared to the reference first quartile after multivariate adjustment for conventional risk factors, indicating that elevated copeptin was a predictor of HF development in older adults.

Currently, copeptin has also been demonstrated to be a relatively good predictor of mortality in patients presenting to the ED with acute dyspnea (187, 199). A cohort study (122) that enrolled 287 patients with acute dyspnea showed that the AUC of copeptin, NT-proBNP, and BNP to predict 30-day mortality was 0.83 (95% CI 0.76–0.90), 0.76 (95% CI 0.67–0.84) and 0.63 (95% CI−0.530.74), respectively. After adjusting for common cardiovascular risk factors and NT-proBNP levels, copeptin was the strongest independent predictor of short-term mortality [HR 3.88 (95% CI 1.94–7.77)]. For those with acute decompensated heart failure (ADHF), copeptin had an HR of 5.99 (95% CI 2.55–14.07) to independently predict short-term mortality.

In addition, for patients with HF, copeptin has been proposed as a potential predictor of outcome or mortality. Copeptin was reported to be associated with left ventricular dysfunction, volumes, remodeling, and clinical HF post-AMI, indicating that copeptin measurement might add up prognostic information (200). The first study of the prognostic performance of copeptin in HF was performed in 2006 by Stoiser et al., who investigated the ability of copeptin and BNP to predict death and re-hospitalization and found that copeptin served as an independent predictor that was superior to BNP in patients with advanced HF (multivariate analysis of combined endpoint: copeptin [chi(2) = 20] vs. BNP [chi(2) = 4.9)] (117). Similar conclusions have been drawn and the prognostic value of copeptin in patients with HF has been widely discussed and confirmed (125, 141, 201–203).

For patients with acute HF, a multicenter trial reported significantly increased the 90-day mortality, readmissions, and ED visits in patients with elevated copeptin levels, especially in those with hyponatremia (HR = 7.36) (125). Another study found that increased copeptin concentrations predicted mortality in acute HF (HR = 1.61) and acute exacerbation of chronic obstructive pulmonary disease (AECOPD) (HR = 1.72), and copeptin reclassified a significant proportion of patients into a more accurate risk stratification in acute HF [0.39 (95% CI 0.06–0.71)] and AECOPD [NRI 0.60 (95% CI 0.19–1.02)] (135). These findings indicated the reliable value of copeptin in the prediction and prognosis of patients with acute HF. For patients with chronic HF, a long-term observational study found that increased copeptin levels correlated with excess mortality irrespective of the clinical signs of disease severity (119). Copeptin has also been demonstrated to be a predictor of long-term mortality in patients with chronic HFrEF (132).

Furthermore, the combination of copeptin with other biomarkers (such as hs-cTnT and NT-proBNP) might improve the prognostic performance of patients with HF compared to a single marker (124, 127). In a prospective study (137), the capacity of mid-regional pro-adrenomedullin (MR-proADM), copeptin, and interleukin-6 were combined with conventional clinical markers to predict the 30-day mortality of patients with acute HF was investigated. The AUC of the clinical model plus copeptin and NT-proBNP was 0.75 (95% CI 0.67–0.83), which was better than the clinical model alone [AUC 0.67 (95% CI 0.58–0.76)]. In summary, copeptin, in combination with other biomarkers, might benefit the monitoring of disease severity and predict the prognosis of HF. Table 4 shows articles on the prognostic value of copeptin in prediction of the outcome of HF.

**Table 4:** Articles about the prognostic value of copeptin (alone or with BNP/NT-proBNP) in predicting the outcome of HF.

**References**	**Sample size**	**Mean/median follow-up duration**	**Selected cutoff value and corresponding prognostic performance**	**Multivariate analysis**	**Main conclusion**
Stoiser et al. (117)	268 with advanced HF	15·8 months	Copeptin 18.3 pmol/L AUC 0.672 BNP 448 pg/mL AUC 0.662	Independent predictors: copeptin (χ^2^ = 4·2, *P* < 0·05), BNP (χ^2^ = 18, *P* < 0.0001), age (χ^2^ = 11·8, *P* < 0.001).	Copeptin is an excellent predictor of outcome in advanced HF patients. Its value is superior to that of BNP in predicting death and a combined endpoint.
Gegenhuber et al. (118)	137 with acute destabilized HF	365 days	Copeptin [AUC 0.688 (0.603–0.764)]−15 pmol/L: sensitivity 85% (71–94), specificity 42% (32–52), PPV 38%, NPV 87%;−45 pmol/L: sensitivity 56% (40–72), specificity 76% (66–84), PPV 50%, NPV 80% BNP [AUC 0.716 (0.633–0.790)]−495 ng/L: sensitivity 83% (68–93), specificity 41% (31–51), PPV 37%, NPV 85%;−1,250 ng/L: sensitivity 56% (40–72), specificity 76% (66–84), PPV 50%, NPV 80%	Copeptin risk ratio 2.62 (1.40–4.92), BNP risk ratio 2.07 (1.07–4.03).	MR-proANP, MR-proADM, and Copeptin measurements might have similar predictive properties compared with BNP determinations for one-year all-cause mortality in acute destabilized HF.
Neuhold et al. (119)	786 HF	15.8 months	Copeptin (AUC 0.711) BNP (AUC 0.711) copeptin + BNP (AUC 0.744)	NA	Increased levels of copeptin are linked to excess mortality, and this link is maintained irrespective of the clinical signs of severity of the disease. Copeptin was superior to BNP or NT-proBNP in this study, but the markers seem to be closely related.
Voors et al. (120)	224 with HF	33 months	Copeptin (AUC 0.81) 25.9 pmol/L: sensitivity 67.7%, specificity 82.5%, PPV 39.6%, NPV 93.8%; BNP (AUC 0.66) 181 pmol/L: sensitivity 50.0%, specificity 79.2%, PPV 28.6%, NPV 90.5%; NT-proBNP (AUC 0.67) 1,980 pmol/L: sensitivity 53.1%, specificity 79.9%, PPV 30.4%, NPV 91.1%.	Copeptin: HR 1.83 (1.26–2.64) for mortality, and HR 1.35 (1.05–1.72) for the composite cardiovascular endpoint; BNP: HR 1.85 (0.79–4.31) for mortality, and HR 1.67 (0.95–2.93) for the composite cardiovascular endpoint; NT-proBNP: HR 1.30 (0.37–4.58) for mortality, and HR 1.73 (0.75–3.99) for the composite cardiovascular endpoint.	Copeptin is a strong and novel marker for mortality and morbidity in patients with HF after AMI. In this population, the predictive value of copeptin was even stronger than BNP and NT-proBNP.
Smith et al. (121)	Total 5,187 (112 with HF)	14 years	NA	Copeptin HR 1.35 (1.03–1.77); NT-proBNP HR 1.95 (1.63–2.34).	Natriuretic peptides, but not other biomarkers, improve discrimination modestly for both diseases above and beyond conventional risk factors and substantially improve classification for HF.
Potocki et al. (122)	287 with acute dyspnea	30 days	Copeptin [AUC 0.83 (0.76–0.90)] 53 pmol/L NT-proBNP [AUC 0.76 (0.67–0.84)] BNP [AUC 0.63 (0.53–0.74)]	Copeptin HR 3.88 (1.94–7.77) in all patients, HR 5.99 (2.55–14.07) in acute decompensated HF; NT-proBNP HR 2.74 (1.27–5.93) in all patients, HR 2.78 (0.78–10.60) in acute decompensated HF.	Copeptin is a new promising prognostic marker for short-term mortality independently and additive to natriuretic peptide levels in patients with acute dyspnea.
Masson et al. (123)	1,237 with chronic and stable HF	3.9 years	Copeptin [AUC 0.66 (0.63–0.70)] 17.1 pmol/L: sensitivity 0.68, specificity 0.59; NT-proBNP [AUC 0.73 (0.70–0.76)] 1,181 pg/mL: sensitivity 0.71, specificity 0.65.	Copeptin χ^2^ = 87, *P* < 0.0001.	In patients with chronic and stable HF enrolled in a multicentre, randomized, clinical trial, measurement of stable precursor fragments of vasoactive peptides provided prognostic information independent of natriuretic peptides which are currently the best biomarkers for risk stratification.
Alehagen et al. (124)	470 elderly patients with HF	13 years	AUC increased from 0.70 to 0.74 (0.68–0.79) by adding NT-proBNP to clinical examination variables, and increased to 0.76 (0.71–0.82) by adding copeptin to clinical examination variables and NT-proBNP.	fourth quartile of copeptin: HR, 1.70 (1.25–2.31) for all-cause mortality; fourth quartile of NT-proBNP: HR 2.17 (1.60–2.94) for all-cause mortality	Among elderly patients with symptoms of HF, elevated concentrations of copeptin and the combination of elevated concentrations of copeptin and NT-proBNP were associated with increased risk of all-cause mortality.
Maisel et al. (125)	1,641 with acute dyspnea; 557 with acute HF	90 days	Copeptin (AUC 0.662) 135 mEq/L; BNP (AUC 0.608) NT-proBNP (AUC 0.668)	Copeptin (38.5 pmol/L) HR 2.014 (1.065–3.810); NT-proBNP (6,305 ng/L) HR 1.695 (0.887–3.240).	Copeptin was highly prognostic for 90-day adverse events in patients with acute HF, adding prognostic value to clinical predictors, serum sodium, and natriuretic peptides.
Peacock et al. (126)	466 with an ED diagnosis of AHF	90 days	Copeptin (AUC 0.803 for the 14-day mortality); MR-proADM and copeptin had the best AUC 0.818; BNP(AUC 0.484 for the 14-day mortality); NT-proBNP (AUC 0.586).	NA	MR-proADM and copeptin, alone or in combination, may provide superior short-term mortality prediction compared to natriuretic peptides and troponin.
Tentzeris et al. (127)	172 with stable chronic HF	1,301 days	copeptin [AUC 0.72 (0.64–0.80)] 18.9 pmol/L: sensitivity 64%, specificity 74% (to predict the primary endpoint).	Copeptin > 16.4 pmol/L HR 1.62 (0.97–2.71) for outcome prediction; NT-proBNP > 1,809 pg/ml HR 1.90 (1.17–3.07).	Combined use of hs-cTnT and copeptin might predict clinical outcome of patients with chronic stable HF.
Balling et al. (128)	340 with HF	55 months	NA	Copeptin HR 1.4 (1.1–1.9) for hospitalization or death (not independent from NT-proBNP), and HR 1.3 (1.0–1.7) for the combined end point of hospitalization or death.	Plasma copeptin levels predict mortalityin outpatients with chronic HF independently from clinical variables, plasma sodium, and loop diuretic doses. Furthermore, copeptin predicts the combined end point of hospitalization or death independently from NT-proBNP
Miller et al. (129)	187 with class III-IV HF	31 months	NA	Raised copeptin HR 1.86 (0.84–4.12); Raised BNP HR 1.39 (0.78–2.48); Combined increases in MR-proANP and copeptin [HR 9.01 (1.24–65.26)] with cTnT (HR 11.1 [1.52–80.85)].	A strategy of serial monitoring of MR-proANP and, of lesser impact, copeptin, combined with cTnT, may be advantageous in detecting and managing the highest-risk outpatients with HF.
Mason et al. (130)	405 residents (aged 65-100 years)	NA	Copeptin (AUC 0.59) 9.5 pmol/L: sensitivity 55%, NPV 80% BNP (AUC 0.80) 115 pg/mL: sensitivity 67%, NPV 86% 145 pg/mL: sensitivity 76%, NPV 97%; NT-proBNP (AUC 0.78) 760 pg/mL: sensitivity 62%, NPV 87%. 1,000 pg/mL: sensitivity 73%, NPV 97%.	NA	No test, individually or in combination, adequately balanced case finding and rule-out for HF in this population; currently, *in-situ* echocardiography provides the only adequate diagnostic assessment.
Wannamethee et al. (131)	3,870 men aged 60–79 years with no diagnosed HF	11 years	NA	Copeptin HR 1.18 (0.79–1.76); NT-proBNP HR 2.15 (1.88–2.48)	NT-proBNP, but not copeptin significantly improves prediction and risk stratification of HF beyond routine clinical parameters obtained in general practice settings in older men both with and without established CVD.
Pozsonyi et al. (132)	195 consecutive patients with HFREF.	5 years	NT-proBNP [AUC 0.740 (0.670–0.810)]; copeptin [AUC 0.776 (0.712–0.841)].	Copeptin (1-SD increase) HR 1.597 (1.189–2.146); NT-proBNP (1-SD increase) HR 1.368 (1.157–1.618).	Copeptin is an independent long-term prognostic marker in HFREF, with possible clinical relevance for multimarker risk prediction algorithms.
Jackson et al. (133)	628 patients recently hospitalized with decompensated HF	3.2 years	NA	BNP HR 1.27 (1.09–1.47); Copeptin HR 0.99 (0.85–1.15).	The novel biomarkers included in this study added little, if any, incremental prognostic value on their own to a model containing established predictors of mortality.
Zabarovskaja et al. (134)	49 with advanced HF, 13 with one year post-LVAD and 22 with one year post-HTx		NA	Copeptin HR 3.28 (1.66–6.50) for death, LVAD or HTx; NT-proBNP HR 2.01 (1.30–3.14).	Copeptin was elevated in, and independently predicted prognosis in, HF. Copeptin was progressively lower after LVAD and HTx. This suggests that improvement in cardiac output with LVAD and HTx may induce progressively reduced activation of vasopressin, which may be a marker for the beneficial effects of LVAD and HTx.
Winther et al. (135)	314 with acute dyspnea	816 days	Copeptin [AUC 0.71 (0.66–0.77)] and NT-proBNP [AUC 0.85 (0.81–0.89)] for discriminating acute HF from non-HF related dyspnea.	Copeptin HR 1.72 (1.21–2.45) for mortality in AECOPD and HR 1.61 (1.25–2.09) for acute HF; NT-proBNP HR 1.62 (1.27–2.06) for mortality only in acute HF.	Copeptin is a strong prognostic marker in both AECOPD and acute HF, while NT-proBNP concentrations predict mortality only in patients with acute HF. NT-proBNP levels are superior to copeptin levels to diagnose acute HF in patients with acute dyspnea.
Jia et al. (136)	129 with severe acute decompensated HF	90 days	Copeptin [AUC 0.602 ± 0.052 (0.499–0.705)]; 890.0 pg/mL: sensitivity 46.8 (32.1–61.9), specificity 69.5 (58.4–79.2), PPV 46.8 (32.1–61.9), NPV 69.5 (58.3–79.3); NT-proBNP [AUC 0.659 ± 0.048 (0.565–0.753)]; 1,471.5 pg/mL: sensitivity 93.6 (82.5–98.7), specificity 32.9 (22.9–44.2), PPV 44.4 (34.5–54.8), NPV 90.0 (73.1–98.0); Combination [AUC 0.670 ± 0.050 (0.573–0.767)].	Copeptin (≥0.89 ng/ml) RR 1.956 (1.048–3.648); NT-proBNP (≥1,471.50 pg/ml) RR 4.415 (1.357–14.358).	Copeptin has similar predictive properties compared with NT-proBNP regarding adverse events within 90-days in patients with severe acute decompensated HF, but that copeptin may not provide superior 90-day prediction compared to NT-proBNP.
Herrero-Puente et al. (137)	547	30 days	Copeptin [AUC 0.70 (0.62–0.78)] clinical model plus copeptin and NT-proBNP [AUC 0.75 (0.67–0.83)]	Copeptin HR 3.17 (1.91–7.14) for 30-day all-cause mortality; NT-proBNP HR 2.77 (1.43–5.35) for 30-day all-cause mortality.	The combination of a clinical model with copeptin and NTproBNP, which are available in the ED, is able to prognose early mortality in patients with an episode of AHF.
Balling et al. (138)	65		Increased levels of log (copeptin) were associated with a reduced cardiac index (r = 0.65 and *p* = 0.04)	NA	Increased copeptin levels in plasma are associated with hemodynamic parameters obtained at right heart catheterization in patients with HF, in particular-reduced cardiac index. Copeptin could be a useful biomarker for abnormal resting hemodynamics in HF.
Düngen et al. (139)	164 with worsening HF	90 days	Copeptin (AUC 0.72 for 90 days); NT-proBNP (AUC 0.66 for 90 days)	Copeptin at admission χ^2^ = 16.63, C-index = 0.724 for 90 day mortality or rehospitalization; re-measurement at 72 h χ^2^ = 23.48, C-index = 0.718; NT-proBNP χ^2^ = 10.53, C-index = 0.646 for 90 day mortality or rehospitalization; at 48 h χ^2^ = 14.23, C-index = 0.650.	This largest sample of serial measurements of multiple biomarkers in WHF found copeptin at admission with remeasurement at 72 h to be the best predictor of 90 day mortality and rehospitalization.
Welsh et al. (140)	1,853	28 months	NA	Copeptin HR 1.66 (1.35–2.04) for the composite outcome in the top tertile compared to the lowest tertile; NT-proBNP HR 3.96 (3.16–4.98) for the composite outcome.	Once NT-proBNP is included, only hsTnT moderately further improved risk stratification in this group of chronic HF with reduced ejection fraction patients with moderate anemia. NT-proBNP and hsTnT far outperform other emerging biomarkers in prediction of adverse outcome.
Yoshikawa et al. (141)	107 patients hospitalized for HF	4.5 years	NA	Copeptin ≥ 18 pmol/L HR 1.77 (1.04–3.01) for all-cause death/HF. BNP ≥ 1,000 pg/mL HR 0.69 (0.38–1.22).	Copeptin was suggested as a useful marker for predicting long-term clinical outcomes in patients with HF.
Molvin et al. (142)	286 patients hospitalized for newly diagnosed or exacerbated HF	17 months	Copeptin [AUC 0.599 (0.530–0.668)]; NT-proBNP [AUC 0.595 (0.525–0.666)]	Copeptin HR 1.70 (1.22–2.36) for increased mortality; NT-proBNP HR 1.85 (1.17–2.17) for increased mortality; HR 1.43 (1.10–1.87) for risk of re-hospitalization.	Among patients hospitalized for HF, elevated plasma levels of NT-proBNP, MR-proADM, copeptin, and cystatin C are associated with higher mortality after discharge, whereas NT-proBNP is the only biomarker that predicts the risk of rehospitalization due to cardiac causes.
Schill et al. (143)	5,297	11.1 years	NA	Copeptin HR 1.63 (1.20–2.21) for increased risk of developing HF.	Elevated copeptin predicts development of HF in older adults. Copeptin is a risk marker of VP-driven HF susceptibility and a candidate to guide prevention efforts of HF targeting the VP system.
Ozmen et al. (144)	155 consecutive patients with acute signs and symptoms of HF.	90 days	Copeptin [AUC 0.844 (0.753–0.935) for the prediction of adverse events within 90 days[ 34 pmol/L: sensitivity 82.5% (70.7–94.3), specificity 86.1% (79.8–92.4), PPV 67.3% (54.2–80.5), NPV 93.4% (88.7–98.1). NT-proBNP [AUC 0.809 (0.729–0.890)] 5,700 pg/mL: sensitivity 72.5% (58.7–86.3), specificity 76.5% (68.8–84.3), PPV 51.8% (38.7–64.9), NPV 88.9% (82.7–95.1).	Increased copeptin RR 1.051 (1.020–1.083) for adverse events.	Copeptin was found to be a strong, novel marker for predicting CV death or HF-related re-hospitalization in patients with acute decompensated HF.

However, contradictory results that copeptin alone or in combination with other conventional biomarkers has limited clinical value cannot be ignored (130, 204). For instance, a randomized controlled trial found that the HR for the composite outcome for copeptin was 1.66 (95% CI 1.35–2.04), but when copeptin was included in a clinical prediction model (including NT-proBNP), there was no additional improvement for risk stratification in this group of patients with chronic HFrEF (140). The ineffectiveness of copeptin might be attributed to heterogeneity in study design, regional and individual differences, variation in biomarker detection, and cut-off values used.

In addition, it remains unclear whether copeptin can be used to monitor and guide medical therapy for patients with HF, and whether there exists a single cut-off level of copeptin for physicians to decide healthcare allocation in HF. Studies have investigated whether NT-proBNP and copeptin levels have the ability to optimize beta-blocker (BB) up-titration in patients with HF, but they came to inconsistent conclusions in terms of the performance of copeptin (205, 206), indicating the need for further well-designed studies with longer follow-up periods to elucidate the role of copeptin in guiding BB therapy. In terms of the therapeutic means targeting the vasopressin system, a prospective study compared a tolvaptan-based vs. furosemide-based diuretic regimen on short-term clinical responses in hyponatremic acute HF. Plasma copeptin levels increased in the tolvaptan group. The reason for this increase might be that tolvaptan increases serum sodium levels and urine output in HF (196). This phenomenon was consistent with another study (207), which reported that copeptin increased from baseline to week 3 (6.3 vs. 21.9 pmol/L) in tolvaptan-treated patients with autosomal dominant polycystic kidney disease. In addition, patients with higher baseline copeptin levels had a larger tolvaptan treatment effect, and those with larger changes in copeptin after 3 weeks had a better disease outcome (less kidney growth and eGFR decline). However, whether pre-treatment copeptin and treatment-induced change can predict the treatment efficacy of tolvaptan in patients with HF still lacks evidence and requires further investigation. In addition, the treatment effect of V1a antagonists or a combined V1a/V2-receptor blockade, as well as the role of copeptin as a therapy-guiding indicator in patients with HF should be explored in future clinical trials.

## Copeptin in Stroke

Stroke is the second leading cause of CVD burden, resulting in over 6 million deaths in 2013 (2, 208). Stroke can be classified into two types, ischemic stroke (IS) and brain hemorrhage, including intracerebral hemorrhage (ICH) and subarachnoid hemorrhage (SAH) (209, 210). Early identification and prediction of adverse outcomes are crucial for optimized treatment and improved prognosis in patients with stroke.

Stroke conditions serve as acute stressors, activating the HPA axis, which regulates the release of CRH from the hypothalamus. CRH stimulates the release of ACTH from the anterior pituitary. Another stimulated hypothalamic hormone is AVP, which can interact with CRH and lead to the secretion of ACTH (211). It has been shown that cerebral ischemia may increase AVP levels in the plasma of patients with stroke (212, 213) and that V1R, but not V2R, is involved in the pathophysiology of secondary brain damage after focal cerebral ischemia (214). A meta-analysis revealed mean copeptin levels in different groups: stroke and non-stroke groups (19.8 ± 17.4 vs. 9.7 ± 6.6 pmol/L, respectively), good vs. poor outcome groups (12.0 ± 3.6 vs. 29.4 ± 14.5 pmol/L, respectively), and survive vs. non-survive stroke patients (13.4 ± 3.2 vs. 33.0 ± 12.3, respectively) (215), suggesting copeptin may guide the management of stroke patients. Interestingly, no significant difference was observed between the stroke group and stroke mimics (diseases with symptoms frequently seen in patients with stroke but caused by non-cerebrovascular pathogeneses) groups (216), indicating that copeptin could not discriminate between stroke and stroke mimics. The team also found no correlation between copeptin levels and the time from symptom onset, although copeptin levels quickly increased within the first minute after the event. Nonetheless, copeptin was independently associated with an increased risk of incident stroke in older men with diabetes [HR = 2.34 (95% CI 1.04–5.27)] (217), indicating that the vasopressin system might be a therapeutic target with effects on stroke risk in this population. Copeptin has been widely proposed as a prognostic marker for predicting the outcomes of patients with stroke.

### Copeptin in Ischemic Stroke

IS is associated with detrimental and fatal conditions and contributed to 62.4% of all stroke incident cases in 2019 (210). Early risk assessments of disease severity and the prognosis are of great importance for optimized treatment and allocation of medical resources. The National Institutes of Health Stroke Scale (NIHSS) score, which ranges from 0 to 42, is used to predict mortality and functional outcomes in patients with stroke (218). In a prospective observational study, copeptin was assessed for the first time in patients with stroke for the prognostic value (219). It was demonstrated that those with unfavorable outcomes and non-survival had significantly higher copeptin levels on admission, and copeptin seemed to be an independent predictor of functional outcomes and mortality. In addition, adding copeptin to the NIHSS improved the prognostic accuracy for the functional outcome (AUC 0.75–0.79) and mortality (AUC 0.85–0.89). Other studies and meta-analyses also demonstrated that copeptin had a high prognostic performance in IS to predict adverse outcomes and mortality (41, 153, 220–222). Furthermore, in a prospective cohort study, copeptin was reported to improve the prognostic ability of the ABCD2 score for the prediction of IS in 31.2% of patients (223), requiring further studies to validate whether the addition of copeptin to the ABCD2 score can help refine the management of patients with the transient ischemic attack (TIA) and reduce healthcare costs. In 2019, De Marchis et al. (224) proposed a copeptin-based prognostic score (CoRisk score) encompassing copeptin levels, age, NIHSS score, and recanalization therapy. This CoRisk score correctly classified 75% of the patients, with an NRI between the calibrated CoRisk scores with and without copeptin of 46%. Further studies are needed to assess the prognostic accuracy of copeptin in combination with other biomarkers.

Recurrent vascular events after TIA and IS should be carefully monitored and predicted. Greisenegger et al. (225) investigated the value of copeptin in the prediction of long-term risk of vascular events after TIA and IS and reported that copeptin could predict recurrent vascular events (adjusted HR = 1.47), vascular-related death (HR = 0.85), all-cause mortality (HR = 1.75), and recurrent IS (HR = 1.22), particularly in patients with cardioembolic stroke (HR = 1.84). However, the lack of adjustment for additional cardiac indices largely limited the interpretation of the results. Therefore, whether copeptin can serve as an independent predictor of vascular events in patients with stroke requires further evaluation in a large population-based cohort.

### Copeptin in Intracerebral Hemorrhage

ICH is the second most common cause of stroke after IS, but ICH accounts for more disability and mortality and a greater economic burden worldwide (226, 227). In patients with SAH, AVP is elevated in the plasma and cerebrospinal fluid, and V1aR is overexpressed in an experimental model of traumatic brain injury (213, 228, 229). Inhibition of V1aR has been reported to reduce the severity of SAH and prevent rebleeding by blunting the post-hemorrhagic hypertonic response in a rat model (230), indicating a potential approach to treat SAH.

Copeptin was elevated in patients with cerebral infarction (CI), ICH, and SAH compared to healthy controls, but it could not discriminate CI, ICH, and SAH from each other (231). In addition, a meta-analysis (232) in 2017 revealed significantly higher copeptin levels in ICH patients with poor prognosis than in survivors and that high copeptin levels were independently associated with a higher risk of mortality in patients with ICH. The World Federation of Neurological Surgeons subarachnoid hemorrhage scale (WFNS) is an accurate staging system for prognostic prediction after aneurysmal subarachnoid hemorrhage (aSAH) (233). The addition of plasma copeptin concentration significantly improved the predictive performance of WFNS scores for symptomatic cerebral vasospasm (AUC 0.848–0.921) and 6-month poor outcome after aSAH (AUC 0.867–0.940) (234). However, copeptin lacks specificity and can be easily influenced by other stressors, so it is suggested to investigate if it is an independent indicator of the prognosis of ICH and whether it can be used in combination with other biomarkers to achieve an optimal prognostic value in stroke and other life-threatening acute conditions.

## Discussion

Provided that copeptin is secreted in equimolar amounts with AVP and correlates well with AVP release, it can serve as a promising and reliable surrogate of AVP, which is difficult to measure. Copeptin measurements have been shown to play essential roles in harmful CVDs. Copeptin has been reported to be an excellent tool for AMI rapid rule-out when combined with cTn evaluation in patients with potential AMI, as well as for risk stratification and outcome prediction in patients with AMI, HF, and stroke. However, additional larger, well-designed trials are still required to assess the incremental value of copeptin when added to conventional diagnostic or prognostic models of CVD and to evaluate the clinical benefits and applicability of copeptin measurement in routine practice and patient care. Whether copeptin can serve as a treatment indicator in therapies targeting the vasopressin system should be further explored and validated.

## Author Contributions

DM wrote the first draft of the manuscript and prepared the figures. JC prepared the tables. XC and LQ conducted review of the manuscript. All authors contributed to the final revision of the manuscript and approved the submitted version.

## Conflict of Interest

The authors declare that the research was conducted in the absence of any commercial or financial relationships that could be construed as a potential conflict of interest.

## Publisher's Note

All claims expressed in this article are solely those of the authors and do not necessarily represent those of their affiliated organizations, or those of the publisher, the editors and the reviewers. Any product that may be evaluated in this article, or claim that may be made by its manufacturer, is not guaranteed or endorsed by the publisher.
